# The first few days of a SARS-CoV-2 infection viewed at single-cell resolution

**DOI:** 10.1371/journal.pbio.3001217

**Published:** 2021-04-26

**Authors:** Tom Gallagher, Paul B. McCray

**Affiliations:** 1 Department of Microbiology and Immunology, Loyola University Chicago, Maywood, Illinois, United States of America; 2 Department of Pediatrics, University of Iowa, Iowa City, Iowa, United States of America

## Abstract

What transpires soon after inhaling Severe Acute Respiratory Syndrome Coronavirus 2 (SARS-CoV-2), the respiratory virus causing Coronavirus Disease 2019 (COVID-19)? Where does infection begin? What are the features of subsequent virus spread? How might host responses quickly contain infection? Two recently published manuscripts have evaluated infection in primary cultures of well-differentiated cells to address these questions and bring more light on the proviral and antiviral components operating during the initial days after SARS-CoV-2 exposure.

Well before Coronavirus Disease 2019 (COVID-19) advanced to pandemic levels, fundamental questions concerning Severe Acute Respiratory Syndrome Coronavirus 2 (SARS-CoV-2) infection in the human respiratory system were under consideration. Answers to these questions were necessary to form a general understanding of viral pathogenesis and to frame COVID-19 prevention and treatment strategies. Obtaining answers continues to involve great effort, in large part because the human respiratory tract is complex, encompassing nasal passages, large and small airways, bronchioles, and alveoli, each distinctly organized for specialized functions and each comprising several variable cell types. Complex relationships between initial infection and subsequent coordinated antiviral responses take place in the respiratory epithelium, the details of which determine disease outcomes. Despite the challenges, answers have come forward at a remarkable pace. Sensitive assays detecting viral RNA and protein in COVID-19 patient samples revealed sino-nasopharyngeal, airway, and alveolar cell infection. Animal models revealed the accumulation and distribution of SARS-CoV-2 throughout the pulmonary system [1]. Air–liquid interface (ALI) cultures of human nasal, airway, and alveolar epithelial cells have provided tractable ex vivo reflections of the complex human respiratory system, suitable to evaluate infection into respiratory cells from different anatomical regions. From ex vivo infections of ALI cultures, gradients of virus susceptibility were identified, highest in cultures derived from nasal cavities and lowest in those from the distal respiratory tract [2]. These and many other seminal findings helped to frame understanding of virus transmission, dynamics of virus entry and spread in respiratory systems, and resulting late-stage COVID-19.

Now, Ravindra and colleagues [3] and Fiege and colleagues [4] extended this research, focusing with high resolution on the first stages of SARS-CoV-2 infection and the innate immune responses taking place soon thereafter. Using infection onto human bronchial epithelial–derived ALI cultures and subsequent single-cell RNA sequencing (see Fig 1), both groups initially centered on virus tropism and confirmed previous reports that infection is initially localized in ciliated cells. This targeted infection may well be significant to pathogenesis, as reduced ciliary motion impedes clearance of harmful pathogens and toxins from the lower respiratory tract, and loss of infected ciliated epithelia may be an early initiator of the host injury–repair response.

**Fig 1:**
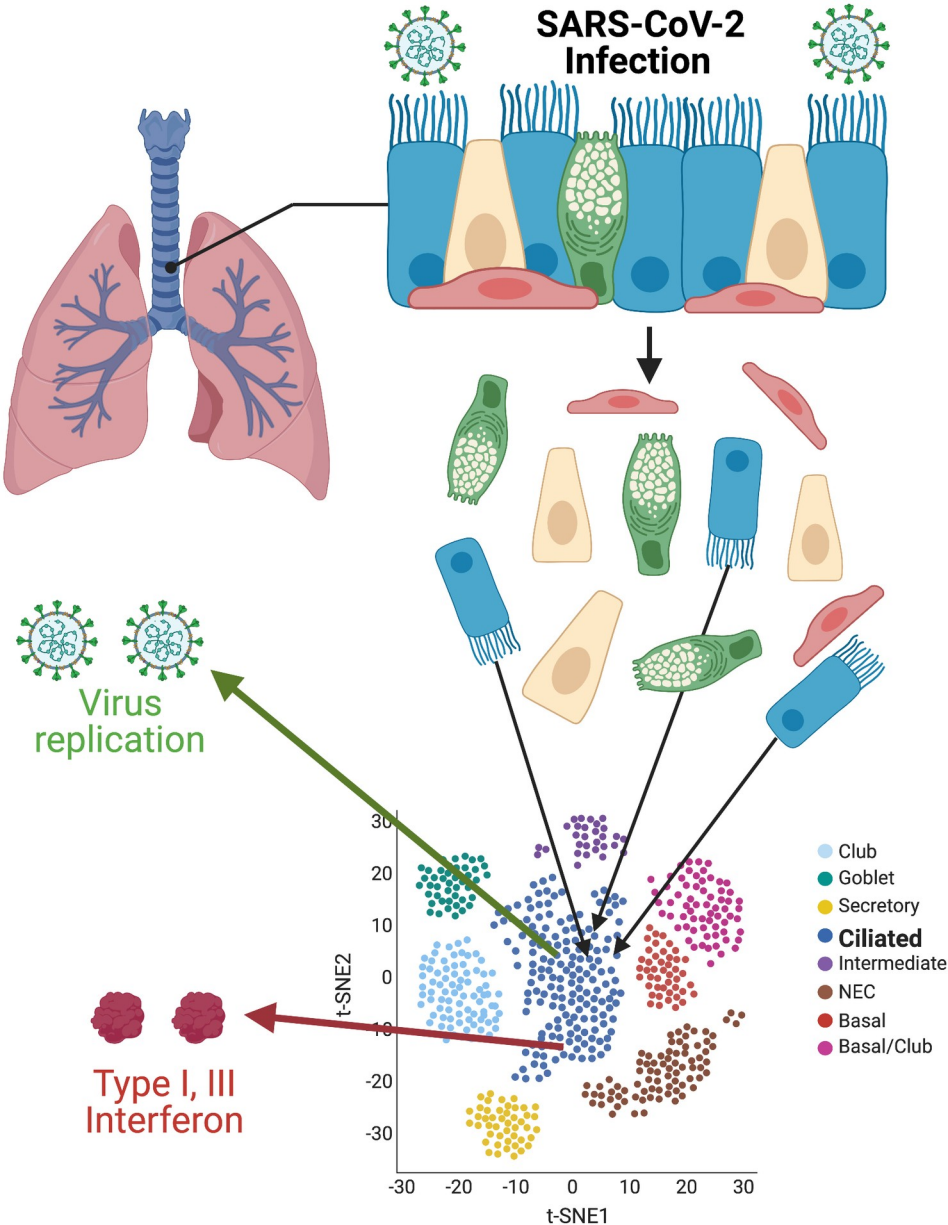
Single-cell resolution of SARS-CoV-2 infection. Primary cultures of well-differentiated bronchial epithelial cells, grown on permeable supports at ALIs, are infected with SARS-CoV-2, then separated for subsequent single-cell RNA sequencing. Using t-SNE statistical methods, cells with similar transcriptome profiles are group-clustered on two-dimensional plots. Individual cells containing SARS-CoV-2 RNAs and antiviral interferon transcripts are identified within clusters. Images were created with BioRender.com. ALI, air–liquid interface; SARS-CoV-2, Severe Acute Respiratory Syndrome Coronavirus 2; t-SNE, t-distributed stochastic neighbor embedding.

Preferential virus attachment to ciliated cells may be explained by the prominent display of angiotensin converting enzyme 2 (ACE2), the SARS-CoV-2 receptor, on distal cilia extensions [5]. This places ACE2 in position to capture viruses flowing through the mucociliary escalator; however, it is important to also note that ACE2 is only one essential determinant conferring virus–cell entry. Host cell proteases are also required to cleave ACE2-bound spike proteins so that they can refold into conformations that catalyze the virus–cell fusion stage of entry. Both Ravindra and colleagues and Fiege and colleagues discovered that one of these virus-activating proteases, transmembrane protease serine 2 (TMPRSS2), was highly expressed in ciliated cells. Fiege and colleagues also demonstrated that camostat, a broad-spectrum inhibitor of TMPRSS2 and related transmembrane serine proteases, potently suppressed infection of ALI cultures. Therefore, in the ALI system, the transmembrane serine proteases are as central to tropism as the primary ACE2 receptors. Whether TMPRSS2 and ACE2 colocalize on the hair-like extensions of ciliated cells is worth considering because such a coincidence at the most accessible apical locations would likely promote highly efficient virus binding, virus–cell fusion, and subsequent infection.

As infection progresses, viruses may spread beyond ciliated cells. Of note, Ravindra and colleagues identified infected basal and club cells as infection continued beyond 1 day. Although Fiege and colleagues did not recognize basal cell infection, and also did not identify TMPRSS2 transcripts in basal cells, the potential for expansive infection deserves further consideration. In the proximal conducting airways, the basal cell is an important progenitor for all the surface epithelial cell types [6]. How the basal cell and its regenerative capacity might be impacted by direct infection, or by paracrine factors coming from neighboring infected cells, is an important issue in recovery from COVID-19, which depends on faithful regeneration of injured airway surfaces.

The Wilen and Langlois teams moved forward from questions of virus tropism to dissect the coordinated innate immune responses to virus infection. The 2 groups identified expected relationships between SARS-CoV-2 RNA accumulation and antiviral interferon transcription, with single-cell resolution revealing that only a small proportion of robustly infected ciliated cells expressed interferons. These few responding cells did, however, produce enough type I and III interferons to elicit pan-ALI expression of interferon-stimulated genes (ISGs). Patterns of ISG expression varied between epithelial cell types, yet overall, ISG products protected bystander cells from infection. Interferons, if sufficiently abundant and present early after infection, will reduce disease in animal models of SARS-CoV [7] and SARS-CoV-2 [8].

In addition to interferons, airway cells release chemokines such as CXCL10 and cytokines including interleukin (IL)-6 and IL-8, which contribute to the early recruitment of adaptive immune cells and onset of inflammation. Hyper-inflammatory responses are a central feature of late-stage COVID-19, with exuberant IL-6 secretion figuring prominently [9]. Ideally, antiviral therapeutics should temper overactive inflammation while preserving antiviral interferon-stimulated effectors. Such pharmacologic manipulation seems possible, as Fiege and colleagues found that infected ALI cultures exposed to the SARS-CoV-2 RNA replication inhibitor remdesevir expressed ISGs but not IL-6. These findings call attention to dosing and timing regimens for direct-acting antiviral drugs, so that infections are suppressed while preserving controlled, appropriate immune responses.

The reports of Ravindra and colleagues and Fiege and colleagues demonstrate the broad utility of ex vivo infection models. The bronchial epithelial cultures they used were valuable in characterizing cell types infected by SARS-CoV-2, cell susceptibility determinants, cytokine induction and signaling, and antiviral therapeutics. Along with complementary models reflecting diffuse alveolar damage and inflammation, the works help to clarify features of lung injury in COVID-19. Longitudinal studies in more sophisticated coculture and animal models will further help unravel the complexities of immune involvement and injury-repair responses that lead to homeostasis or disease chronicity. Taken in the broad context of the thousands of reports on SARS-CoV-2 and COVID-19, the findings in Ravindra and colleagues and Fiege and colleagues add insights into the initial waves of infection and local airway responses. They feed into meta-analyses of single-cell omics data [10] and in doing so, bring out both detailed and holistic maps for understanding SARS-CoV-2 biology and COVID-19 disease. As mass vaccination ends the COVID-19 pandemic, the approaches used by Ravindra and colleagues, Fiege and colleagues, and many others will continue to unravel details of current and future infectious diseases threatening respiratory health.

## Abbreviations

ACE2: angiotensin converting enzyme 2
ALI: air–liquid interface
COVID-19: Coronavirus Disease 2019
IL: interleukin
ISG: interferon-stimulated gene
SARS-CoV-2: Severe Acute Respiratory Syndrome Coronavirus 2
TMPRSS2: transmembrane protease serine 2

## References

[1] Deng W, Bao L, Liu J, Xiao C, Liu J, Xue J, et al. Primary exposure to SARS-CoV-2 protects against reinfection in rhesus macaques. Science. 2020;369(6505):818–23. 10.1126/science.abc5343 32616673 PMC7402625

[2] Hou YJ, Okuda K, Edwards CE, Martinez DR, Asakura T, Dinnon KH III, et al. SARS-CoV-2 Reverse Genetics Reveals a Variable Infection Gradient in the Respiratory Tract. Cell. 2020;182(2):429–46.e14. 10.1016/j.cell.2020.05.042 32526206 PMC7250779

[3] Ravindra NG, Alfajaro MM, Gasque V, Huston NC, Wan H, Szigeti-Buck K, et al. Single-cell longitudinal analysis of SARS-CoV-2 infection in human airway epithelium identifies target cells, alterations in gene expression and cell state changes. PLoS Biol. 2021. 10.1371/journal.pbio.3001143 33730024 PMC8007021

[4] Fiege JK, Thiede JM, Nanda HA, Matchett WE, Moore PJ, Montanari NR, et al. Single cell resolution of SARS-CoV-2 tropism, antiviral responses, and susceptibility to therapies in primary human airway epithelium. PLoS Pathog. 2021;17(1):e1009292. 10.1371/journal.ppat.1009292 33507952 PMC7872261

[5] Lee IT, Nakayama T, Wu CT, Goltsev Y, Jiang S, Gall PA, et al. ACE2 localizes to the respiratory cilia and is not increased by ACE inhibitors or ARBs. Nat Commun. 2020;11(1):5453. 10.1038/s41467-020-19145-6 33116139 PMC7595232

[6] Rock JR, Onaitis MW, Rawlins EL, Lu Y, Clark CP, Xue Y, et al. Basal cells as stem cells of the mouse trachea and human airway epithelium. Proc Natl Acad Sci U S A. 2009;106(31):12771–5. 10.1073/pnas.0906850106 19625615 PMC2714281

[7] Channappanavar R, Fehr AR, Vijay R, Mack M, Zhao J, Meyerholz DK, et al. Dysregulated Type I Interferon and Inflammatory Monocyte-Macrophage Responses Cause Lethal Pneumonia in SARS-CoV-Infected Mice. Cell Host Microbe. 2016;19(2):181–93. 10.1016/j.chom.2016.01.007 26867177 PMC4752723

[8] Dinnon KH III, Leist SR, Schafer A, Edwards CE, Martinez DR, Montgomery SA, et al. A mouse-adapted model of SARS-CoV-2 to test COVID-19 countermeasures. Nature. 2020;586(7830):560–6. 10.1038/s41586-020-2708-8 32854108 PMC8034761

[9] Blanco-Melo D, Nilsson-Payant BE, Liu WC, Uhl S, Hoagland D, Moller R, et al. Imbalanced Host Response to SARS-CoV-2 Drives Development of COVID-19. Cell. 2020;181(5):1036–45.e9. 10.1016/j.cell.2020.04.026 32416070 PMC7227586

[10] Muus C, Luecken MD, Eraslan G, Sikkema L, Waghray A, Heimberg G, et al. Single-cell meta-analysis of SARS-CoV-2 entry genes across tissues and demographics. Nat Med. 2021. 10.1038/s41591-020-01227-z 33654293 PMC9469728

